# Efficacy and safety of denosumab compared with conventional medications in the treatment of osteoporosis: A meta-analysis with trial sequential analysis

**DOI:** 10.1097/MD.0000000000047150

**Published:** 2026-01-09

**Authors:** Liang Li, Jiahao Xu, Hongjian Ji, Shuang Xia, Fengchao Shi

**Affiliations:** a Department of Orthopedics, Affiliated Hospital 6 of Nantong University, Yancheng 224001, China; b School of Pharmacy, Jiangsu Medical College, Yancheng City Therapeutic Drug Precision Testing Engineering Center, Yancheng 224005, China.

**Keywords:** adverse events, conventional medications, denosumab, osteoporosis, trial sequential analysis

## Abstract

**Background:**

This meta-analysis aimed to systematically evaluate the efficacy and safety of denosumab versus conventional medications in treating osteoporosis.

**Methods:**

Randomized controlled trials from PubMed, ScienceDirect, Web of Science, CNKI, WanFang Data, and VIP were searched via the web from the onset to April 2025. RevMan 5.4 software was used for performing a systematic review. Trial sequential analysis (TSA) of serious adverse events (SAEs) was performed using TSA 0.9.5.10 Beta.

**Results:**

A total of 15 studies with 16,437 patients were included. Compared to the control group (conventional medications), the experimental group with denosumab showed no significant difference in increasing total hip bone mineral density (BMD) (mean difference [MD] = 0.03, 95% confidence interval [CI] = [0.00–0.06], *P* = .06) and femoral neck BMD (MD = 0.02, 95% CI = [−0.04 to 0.08], *P* = .43), but it significantly improved lumbar spine BMD (MD = 0.07, 95% CI = [0.03–0.11], *P* = .003). In addition, there were no differences between the 2 groups in terms of reducing osteonecrosis of the jaw (odds ratio [OR] = 1.49, 95% CI = [0.65–3.46], *P* = .35), atypical fractures (OR = 1.77, 95% CI = [0.38–8.31], *P* = .47), AEs (OR = 0.98, 95% CI = [0.91–1.07], *P* = .71), and SAEs (OR = 0.94, 95% CI = [0.53–1.69], *P* = .85), and TSA showed an insufficient information size with SAEs of denosumab compared with conventional medications in the treatment of osteoporosis.

**Conclusion:**

It can be concluded that denosumab can improve lumbar spine BMD compared with conventional medications for osteoporosis; furthermore, no evidence was found that the denosumab had a significantly higher incidence of SAEs than the control group and it is safe to apply. However, given the potential bias of the included studies, more high-quality, large-sample randomized controlled trials are needed to validate this conclusion further and to provide a more reliable basis for the use of medication in the clinical treatment of osteoporosis. The registration number in PROSPERO is CRD420251051061 (http://www.crd.york.ac.uk/PROSPERO/).

## 1. Introduction

Osteoporosis is a subclinical disease associated with age and gender, characterized by decreased bone mass and microarchitectural deterioration, which ultimately leads to decreased bone strength and increased fracture risk.^[1,2]^ Osteoporosis is a common cause of spine and hip fractures, and the number of elderly people disabled or even killed by these fractures continues to increase each year, posing a significant threat and burden to the global economy and human health.^[3,4]^ Besides, osteoporotic fractures also affect postmenopausal women, severely impairing patient autonomy, functionality, and quality of life.^[5]^ Therefore, medications to prevent or treat osteoporosis and reduce the burden on human health are critically important.

Denosumab and bisphosphonates such as alendronate and risedronate are the most commonly used medications in the treatment of osteoporosis worldwide.^[6,7]^ Receptor activator of nuclear factor-κB ligand (RANKL) is a cytokine crucial for the survival and development of osteoclasts, so inhibitory RANKL can prevent osteoclast differentiation and enhance bone mass.^[8]^ Denosumab, a monoclonal antibody against RANKL, is an anti-bone resorption drug that inhibits osteoclast differentiation and function.^[9]^ Its mechanism of action is to bind to the unique ring structure of RANKL, preventing its interaction with nuclear factor κ B receptor activator (RANK), which is expressed on the cell membrane surface of osteoclasts. By obstructing RANKL, it then induces a reduction in bone resorption and increases bone density by blocking osteoclast formation, function, and survival.^[10]^

Several systematic reviews comparing the efficacy and safety of denosumab and bisphosphonates have been published over the past years.^[3,11]^ and it has been shown to increase bone density in the hip, lumbar spine, femoral neck, and other sites, unlike bisphosphonates. However, there are some shortcomings: They only compared the efficacy of denosumab with bisphosphonates in osteoporosis and not with other medications. Furthermore, osteonecrosis of the jaw (ONJ) and atypical fractures, as potential complications of denosumab therapy, have long been a focus of clinical concern. Nevertheless, their causal association with the drug remains controversial, and few studies have systematically assessed them as primary endpoints to date.^[12]^ The present study conducted a meta-analysis with TSA to fill this gap and a large sample size to critically assess the efficacy and safety of denosumab compared to conventional medications for the treatment of postmenopausal osteoporosis.

## 2. Materials

### 2.1. Registration

A predetermined, written protocol of this overview was registered in the International Prospective Register of Systematic Overview (PROSPERO) database (https://www.crd.york.ac.uk/PROSPERO/), registration number: CRD420251051061. As this was a meta-analysis, permission from an ethical committee or institutional review board was not required. This article follows the Preferred Reporting Items for Systematic Evaluation and Meta-Analysis (PRISMA) guidelines.^[13]^

### 2.2. Data sources and search strategies

Considering the coverage of journals and accessibility of resources, this meta-analysis conducted a systematic search up to April 2025 in PubMed, China Knowledge Network (CNKI), Wanfang database, VIP database, ScienceDirect and Web of Science. The search strategy was developed in collaboration with all authors, Among the search tactics utilized by the English Databases were ((AMG 162[Title/Abstract]) OR (Xgeva[Title/Abstract]) OR (Prolia[Title/Abstract]) OR(Denosumab[Title/Abstract])) AND ((Osteoporoses[Title/Abstract]) OR (Osteoporosis, Age-Related[Title/Abstract]) OR (Osteoporosis, Age Related[Title/Abstract]) OR (Age-Related Osteoporosis[Title/Abstract]) OR (Age-Related Osteoporoses[Title/Abstract]) OR (Age Related Osteoporosis[Title/Abstract]) OR (Osteoporoses, Age-Related[Title/Abstract]) OR(Bone Loss, Age-Related[Title/Abstract]) OR (Age-Related Bone Loss[Title/Abstract]) OR (Age-Related Bone Losses[Title/Abstract]) OR (Bone Loss, Age Related[Title/Abstract]) OR (Bone Losses, Age-Related[Title/Abstract]) OR (Osteoporosis, Senile[Title/Abstract]) OR (Osteoporoses, Senile[Title/Abstract]) OR (Senile Osteoporoses[Title/Abstract]) OR (Senile Osteoporosis[Title/Abstract]) OR (Osteoporosis, Involutional[Title/Abstract]) OR (Osteoporosis, Post-Traumatic[Title/Abstract]) OR (Osteoporosis, Post Traumatic[Title/Abstract]) OR (Post-Traumatic Osteoporoses[Title/Abstract]) OR (Post-Traumatic Osteoporosis[Title/Abstract]) OR (osteoporosis[Title/Abstract])) AND ((Randomized Controlled Trial[Title/Abstract]) OR (RCT[Title/Abstract])). The Chinese databases were searched using textual phrases such as ([“di shu dan kang (which means denosumab in Chinese)”]) AND ([“gu zhi shu song (which means osteoporosis in Chinese)”]) AND ([“sui ji dui zhao shi yan (which means RCTs in Chinese)”]). In addition, reference lists of relevant studies, reviews and meta-analyses were manually checked for additional literature.

### 2.3. Study selection and eligibility criteria

Two independent reviewers examined the titles and abstracts of all admitted publications, followed by a full-text assessment to determine eligibility. A senior reviewer resolved any disagreements. Trials that met the following criteria based on the Participants, Interventions, Comparisons, and Outcomes (PICO) methodology were included: (P) patients with primary osteoporosis; (I) denosumab; (C) other medications for the treatment of osteoporosis (including alendronate, ibandronate, teriparatide, or romosozumab, among others); and (O) bone mineral density (BMD), adverse events (AEs), and others. The following articles were excluded from this article: randomized controlled trials (RCTs) without target populations, such as patients with secondary osteoporosis (e.g., cancerous osteoporosis and glucocorticoid osteoporosis) and musculoskeletal pain; RCTs without an aiming outcome; RCT sub-studies or post hoc analyses, etc; and non-RCTs (e.g., review articles, case reports, commentaries, editorials).

### 2.4. Quality assessment

This paper utilized the Cochrane Consumers and Communication Review Group’s Study Quality Guide to evaluate the quality of the studies. The assessment focused on the following areas: random sequence generation (to address selection bias), allocation concealment (to prevent selection bias), blinding of participants and personnel (to mitigate implementation bias), blinding of outcome assessment (to reduce measurement bias), incomplete outcome data (to identify follow-up bias), selective reporting (to address reporting bias), and other potential biases.

### 2.5. Data extraction

A PRISMA flowchart was used to guide the screening of featured studies. The retrieved literature was then imported into Endnote X9 software. Initial screening of titles and abstracts of eligible studies was conducted by 2 authors initially and independently, with disagreements resolved by senior reviewers. Data were gathered using a standardized extraction form and a systematic extraction procedure, which included the following elements: study characteristics (first author, year of publication, country of study); participant characteristics (data on study design, number of subjects, mean age (years), dosage, duration of treatment (months), and AEs of denosumab and control medication); reference criteria (diagnostic criteria and prevalence of osteoporosis); indicator tests (osteoporosis risk assessment tools and their thresholds); raw outcome data (number of true positives, false positives, true negatives, and false negatives). If necessary, the authors were contacted to verify information and obtain additional details. For dose discovery studies, we chose the most commonly used doses in clinical practice to facilitate relevant comparisons.

### 2.6. Statistical analysis

The RevMan software program (version 5.4; The Cochrane Collaboration, Oxford, UK) was used.^[14]^ Then, chi-square (χ^2^) was used to test for heterogeneity, and a fixed-effects model was used if *P* ≥ .1 or I^2^ ≤ 50%, and a random-effects model was used if *P* < .1 or I^2^ > 50%. For continuous outcomes (lumbar spine, total hip BMD), mean difference (MD) was used as the effect measure. For dichotomous outcomes (ONJ, atypical fractures, AEs, and SAEs), risk ratios were used. 95% confidence intervals (CIs) were calculated for each outcome.

Finally, trial sequential analysis (TSA) of the SAEs was performed, TSA overcomes the shortcomings of traditional meta-analysis, reduces false positive results due to random errors, and allows estimation of the sample size required for Meta-analysis to improve the credibility of this study.

## 3. Results

### 3.1. Description of studies

**Figure 1.**
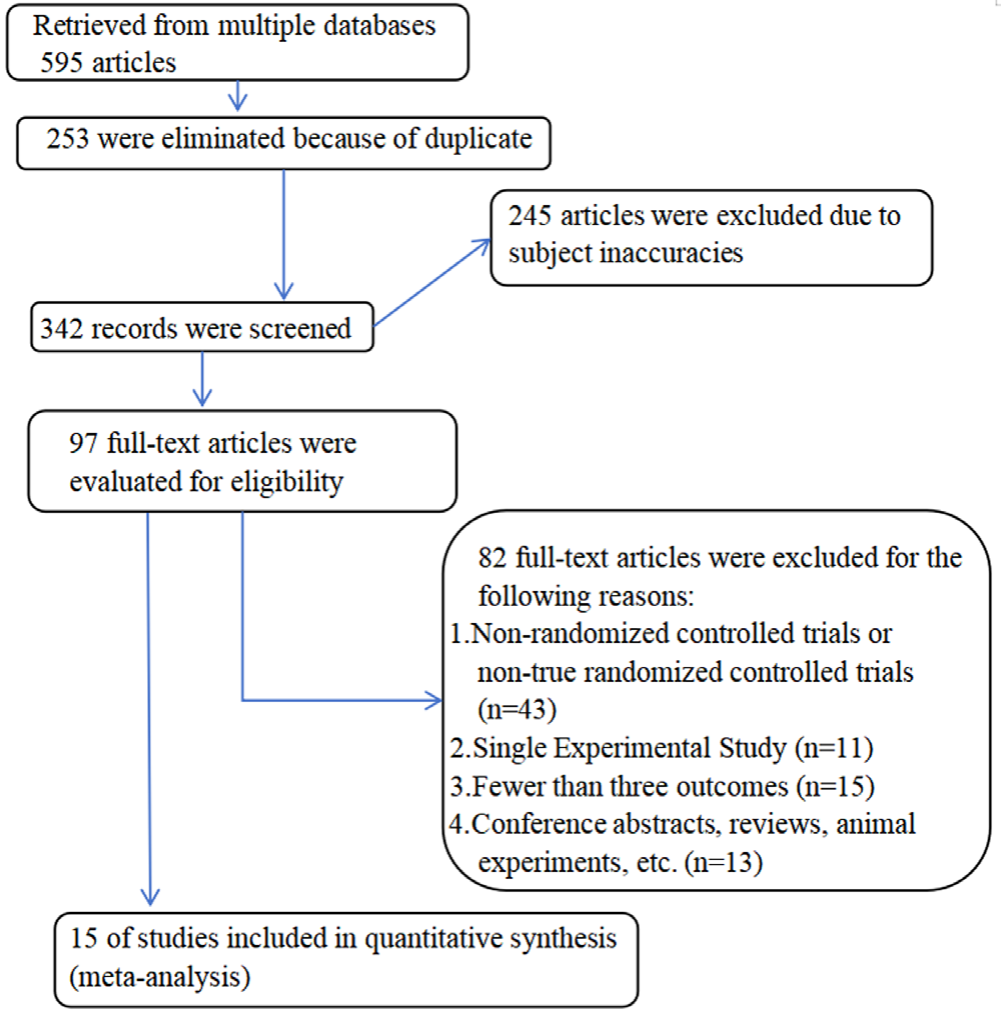
Flowchart of study selection.

The study selection process is shown in the flow chart in Figure 1. Initially, 595 records were identified through database searches and manual searches, 253 were excluded after removing duplicates, and 245 of the remaining 342 were excluded from title and abstract screening because they did not meet the inclusion criteria. The full text of the remaining 97 studies was assessed, of which 82 were excluded for various reasons: 1. non-RCTs or non-truly RCTs (n = 43); 2. single-experiment studies (n = 11); 3. fewer than 3 outcome metrics (n = 15); and 4. abstracts of conferences, reviews, and animal experiments, etc (n = 13). In the end, a total of 15 studies were enrolled^[15–29]^ (where Bone et al and Papapoulos et al present different phases of the same trial, with the former presenting the results of the fifth year, and the latter presenting the results of the tenth year). Consensus was reached by all authors involved in the selection and evaluation process.

### 3.2. Basic characteristics of the included studies

Table 1 summarizes the features of all the randomized controlled trials published between 2006 and 2025. These studies involved 16,437 people, with 8506 assigned to the trial group and 7931 to the control group. All studies evaluated the effect of denosumab in the treatment of primary osteoporosis, administered at a dosage of 60 mg subcutaneously every 6 months. Specifically, 3 studies were conducted in China, 7 in the United States, 3 in Japan, and one each in the United Kingdom and Saudi Arabia. Out of the 15 studies, 6 used a placebo as the control group, while 2 each compared denosumab to bisphosphonates such as alendronate, zoledronic acid, and ibandronic acid. Additionally, there was one study each comparing denosumab to romosozumab, teriparatide, and the generic version of denosumab, CT-P41. All studies had a minimum duration of 12 months.

**Table 1:** The sum of the specific characteristics of the included literatures.

Author	Study		Sample		Intervention	Comparison	Mean age		Duration	Adverse event		Serious adverse event		Osteonecrosis of the jaw		Atypical fracture	
		Country	Intervention	Comparison			Intervention	Comparison	(month)	Intervention	Comparison	Intervention	Comparison	Intervention	Comparison	Intervention	Comparison
Bone 2017^[15]^	RCT	USA	2343	2207	Denosumab (60 mg q6m)	Placebo (3 yr) + denosumab (60 mg q6m 7y)	74.9 ± 5.0	74.8 ± 5.1	120	\	\	\	\	7	6	1	1
Cosman 2016^[16]^	RCT	USA	3589	3591	Romosozumab (210 mg) + denosumab (60 mg q6m 1y)	Placebo (1 yr) + denosumab (60 mg q6m 1y)	70.9 ± 7.0	70.8 ± 6.9	24	3053	3069	565	540	2	0	1	0
Jiang 2024^[17]^	RCT	China	329	111	Denosumab (60 mg q6m)	Placebo	66.05 ± 5.79	64.93 ± 6.23	12	239	84	31	16	\	\	\	\
Kendler 2010^[18]^	RCT	USA	253	251	Denosumab (60 mg q6m)	Alendronate (70 mg qw)	66.9 ± 7.8	68.2 ± 7.7	12	197	196	15	16	\	\	\	\
Kobayakawa 2021^[19]^	RCT	Japan	69	69	Denosumab (60 mg q6m)	Romosozumab (210 mg qm)	73.1 ± 12.3	76.3 ± 8.7	12	6	25	0	1	\	\	\	\
Mcclung 2006^[20]^	RCT	USA	47	47	Denosumab (60 mg q6m)	Alendronate (70 mg qw)	63.1 ± 8.1	62.8 ± 8.2	12	39	42	18	1	\	\	\	\
Miller 2016^[21]^	RCT	USA	321	322	Denosumab (60 mg q6m)	Zoledronic acid (5 mg qy)	68.5 ± 7.1	69.5 ± 7.7	12	199	199	25	29	\	\	2	1
Papapoulos 2012^[22]^	RCT	UK	2343	2207	Denosumab (60 mg q6m)	Placebo (3 yr) + denosumab (60 mg q6m 2y)	74.9 ± 5.0	74.8 ± 5.1	60	1955	1826	442	428	2	0	0	0
Recknor 2013^[23]^	RCT	USA	417	416	Denosumab (60 mg q6m)	Ibandronate (150 mg qm)	67.2 ± 68.1	66.2 ± 67.8	12	245	230	39	22	\	\	\	\
Reginster 2024^[24]^	RCT	KSA	239	240	Denosumab (60 mg q6m)	CT-P41 (60 mg q6m)	66	66	12	167	181	10	7	1	0	0	0
Sugimoto 2014^[25]^	RCT	Japan	404	406	Denosumab (60 mg q6m)	Placebo + denosumab (60 mg q6m 1y)	71.5 ± 7.29	70.8 ± 7.63	36	343	339	9	8	0	1	0	0
Kobayakawa 2022^[26]^	RCT	Japan	62	62	Denosumab (60 mg q6m)	Ibandronate (100 mg qm)	74.3 ± 9.3	74.9 ± 8.2	12	5	10	0	0	\	\	\	\
Tsai 2013^[27]^	RCT	USA	33	31	Denosumab (60 mg q6m)	Teriparatide (20 μg qd)	66.3 ± 8.3	65.5 ± 7.9	12	0	0	\	\	\	\	\	\
Zhang 2022^[28]^	RCT	China	337	118	Denosumab (60 mg q6m)	Placebo	66.0 ± 6.82	67.5 ± 6.55	12	273	93	29	17	\	\	\	\
Zhou 2025^[29]^	RCT	China	63	60	Denosumab (60 mg q6m)	Zoledronic acid (5 mg qy)	66.69 ± 7.58	67.75 ± 7.22	12	7	5	\	\	\	\	\	\

RCT = randomized controlled trial.

### 3.3. Quality assessment of inclusion in RCTs

**Figure 2.**
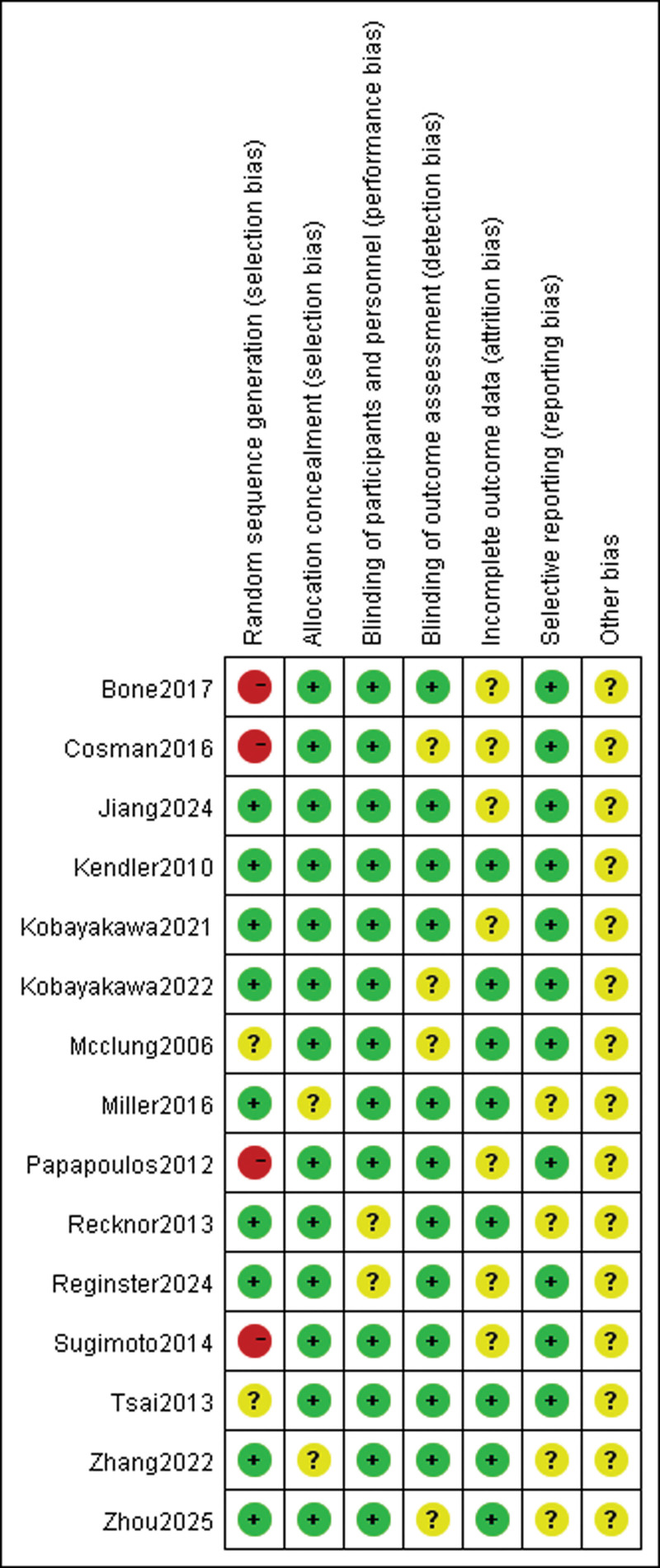
Risk of bias summary.

**Figure 3.**
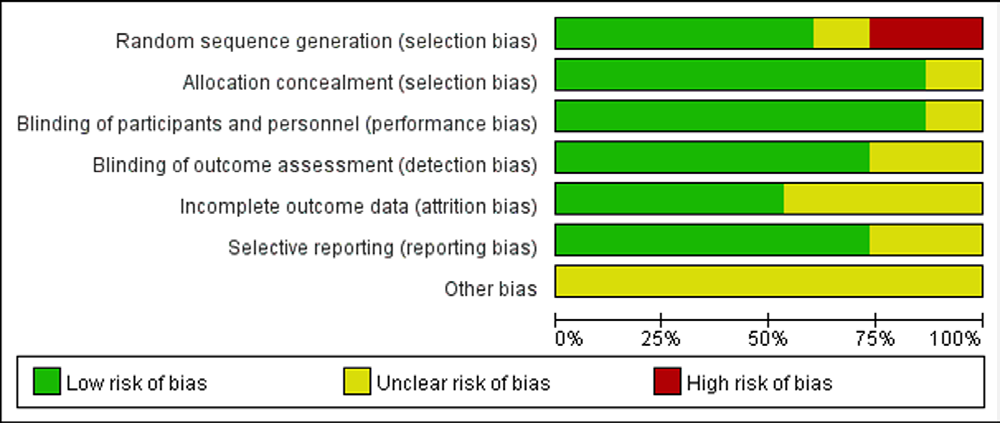
Risk of bias graph.

The Cochrane Risk of Bias Tool was adopted to evaluate the study’s risk of bias. The higher the risk of publication bias, the more likely it is to lead to problems such as decreased stability of results, which in turn can mislead clinical decision-making and policy-making. Of the 15 articles included, the number of articles meeting the criteria ranged from 4/7 to 6/7 per article based on the tool’s assessment criteria. Specifically, 9 of the included RCTs explicitly documented the specific methodology used to generate the randomized sequence. However, 4 of the remaining 6 studies were high-risk because all subjects were taking the same drug. In addition, 13 articles mentioned circumstances related to allocation concealment. In terms of blinding implementation, 13 studies were reported, and the blinding status of the remaining studies was unclear. 11 RCTs met the criterion of completeness of outcome data. All included studies described baseline comparisons and documented the informed consent process of participants; however, 6 studies had a high number of participants lost to follow-up. In particular, in all included studies, all other biases were categorized as unclear risk of bias because we did not know whether the funding agency was involved in the trial. Detailed information on the risk of bias assessment for each trial is provided in Figures 2 and 3.

### 3.4. Results of meta-analysis

#### 3.4.1. ONJ

**Figure 4.**
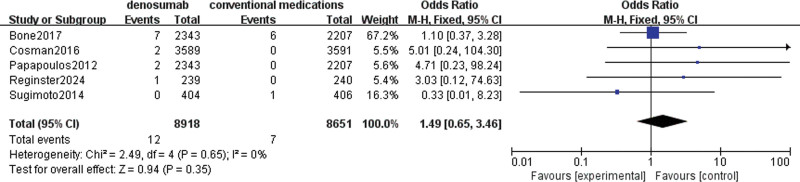
Meta-analysis of denosumab versus conventional medications group at osteonecrosis of the jaw.

A total of 5 studies (two of which were different phases of the same trial) involving 13,019 patients were used to evaluate denosumab versus conventional medications in terms of increased risk of ONJ. The outcome data showed no significant heterogeneity (I^2^ = 0%), so a fixed-effects model was chosen. There was no statistically significant difference in the incidence of ONJ with denosumab compared to conventional medications (OR = 1.49, 95% CI = [0.65–3.46], *P* = .35), as presented in Figure 4.

#### 3.4.2. Atypical fractures

**Figure 5.**
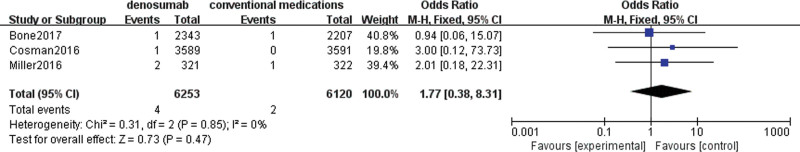
Meta-analysis of denosumab versus conventional medications group at atypical fracture.

A total of 3 studies involving 12,373 patients were used to evaluate denosumab versus conventional medications in terms of increased risk of atypical fractures. The data showed no significant heterogeneity (I^2^ = 0%), so a fixed-effects model was chosen. There was no statistically significant difference in the incidence of atypical fractures with denosumab compared with conventional medications (OR = 1.77, 95% CI = [0.38–8.31], *P* = .47), as shown in Figure 5.

#### 3.4.3. AEs

**Figure 6.**
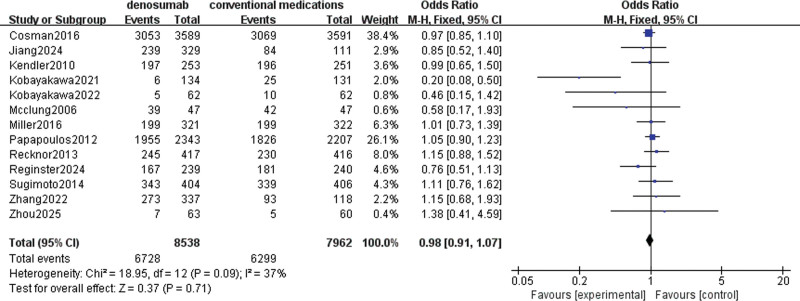
Meta-analysis of denosumab versus conventional medications group at AEs. AE = adverse event.

A total of 13 studies involving 16,373 patients were used to evaluate denosumab versus conventional medications in terms of increased risk of AEs. I^2^ = 37%, there was no significant heterogeneity, so a fixed effect model was chosen. There was no statistically significant difference in increased AEs with denosumab compared to conventional medications (OR = 0.98, 95% CI = [0.91–1.07], *P* = .71), as illustrated in Figure 6.

#### 3.4.4. SAEs

**Figure 7.**
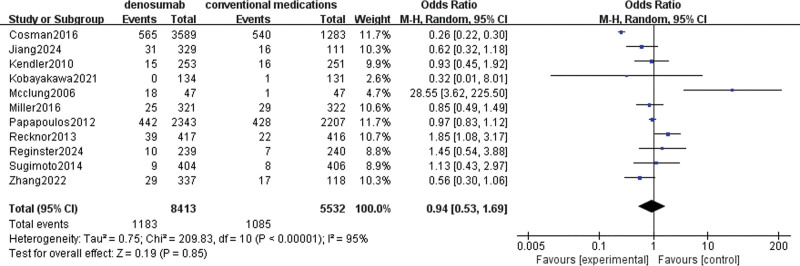
Meta-analysis of denosumab versus conventional medications group at SAEs. SAE = serious adverse event.

A total of 12 studies involving 16,250 patients were used to evaluate denosumab versus conventional medications in terms of increased risk of SAEs. Pooled data showed high heterogeneity (I^2^ = 95%), so a random effects model was chosen. The data showed little to no difference in increased risk of SAEs between denosumab and conventional medicines (OR = 0.94, 95% CI = [0.53–1.69], *P* = .85), as shown in Figure 7.

#### 3.4.5. Lumbar BMD

**Figure 8.**
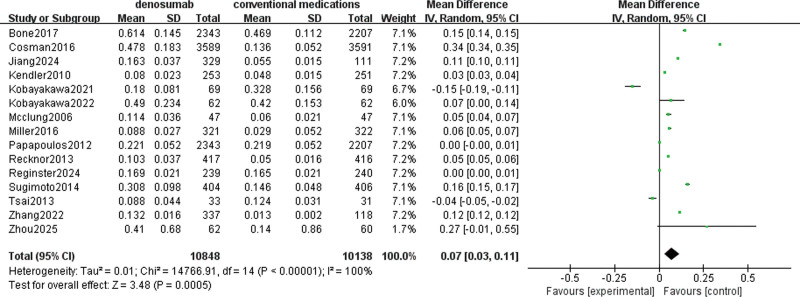
Meta-analysis of denosumab versus conventional medications group at lumbar spine BMD. BMD = bone mineral density.

A total of 15 studies involving 16,437 patients were used to evaluate denosumab versus conventional medications in increasing BMD of lumbar spine. The pooled data showed high heterogeneity (I^2^ = 100%), so a random effects model was chosen. There was a significant difference in increasing BMD of lumbar spine with denosumab compared to conventional medications (MD = 0.07, 95% CI = [0.03–0.11], *P* = .003), as depicted in Figure 8.

#### 3.4.6. Hip BMD

**Figure 9.**
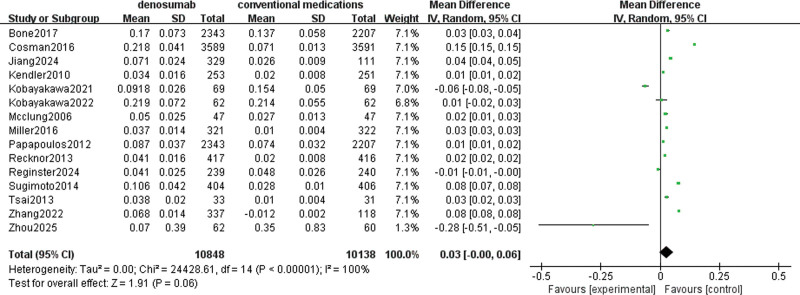
Meta-analysis of denosumab versus conventional medications group at total hip BMD. BMD = bone mineral density.

A total of 15 studies involving 16,437 patients were used to evaluate denosumab versus conventional medications in increasing BMD of the total hip. The pooled data showed high heterogeneity with I^2^ = 100%, so a random-effects model was chosen. There was no significant difference in increasing BMD of lumbar spine with denosumab compared with conventional medications (MD = 0.03, 95% CI = [0.00–0.06], *P* = .06), as shown in Figure 9.

#### 3.4.7. Femoral neck BMD

**Figure 10.**
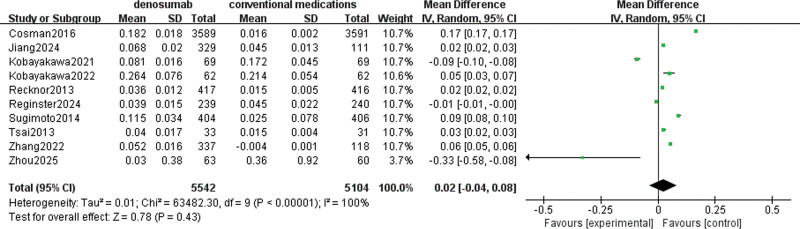
Meta-analysis of denosumab versus conventional medications group at femoral neck bone density.

A total of 10 studies involving 10,646 patients were used to evaluate denosumab versus conventional medications in increasing femoral neck density. The pooled data showed high heterogeneity with I^2^ = 100%, so a random effects model was chosen. There was no significant difference in increasing femoral neck BMD with denosumab compared with conventional medications (MD = 0.02, 95% CI = [−0.04 to 0.08], *P* = .43), as depicted in Figure 10.

### 3.5. Subgroup analysis for Lumbar BMD

**Figure 11.**
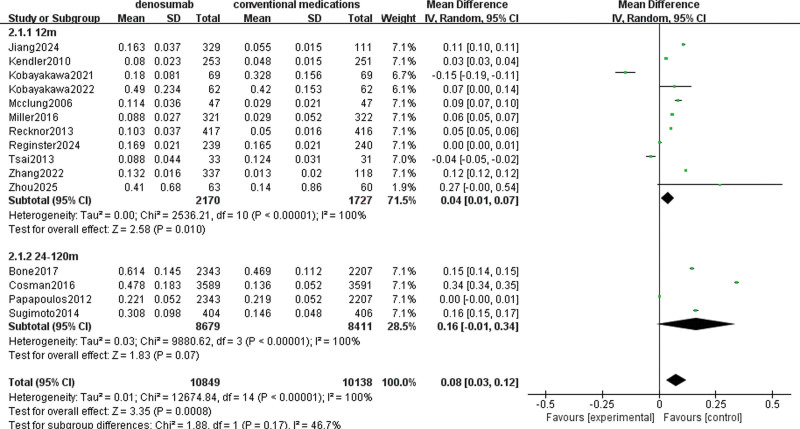
Subgroup analysis of denosumab group versus conventional medications group at lumbar spine BMD in 2 different periods. BMD = bone mineral density.

Given the high degree of heterogeneity detected in the outcome metrics of BMD of the lumbar spine, subgroup analyses were performed by dividing the groups based on differences in medication duration. The analysis results are shown in Figure 11. Groups 1 and 2 represent studies with medication durations of 12 months and 24 to 120 months, respectively. There is a high degree of heterogeneity between the groups; however, the difference is statistically significant, indicating that medication duration may be a source of heterogeneity in increasing the BMD of the lumbar spine. In the future, consideration is given to planning additional subgroup analyses to explore possible sources of high heterogeneity in other outcome metrics.

### 3.6. Publication bias

**Figure 12.**
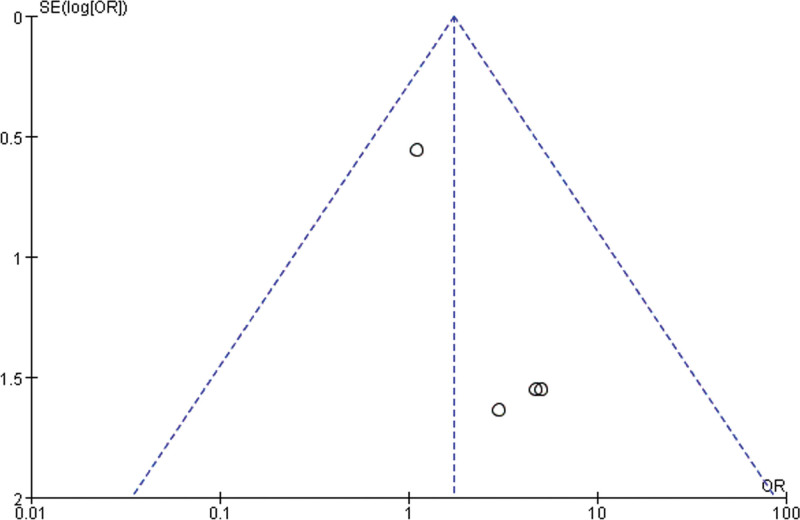
Funnel plot of osteonecrosis of the jaws.

**Figure 13.**
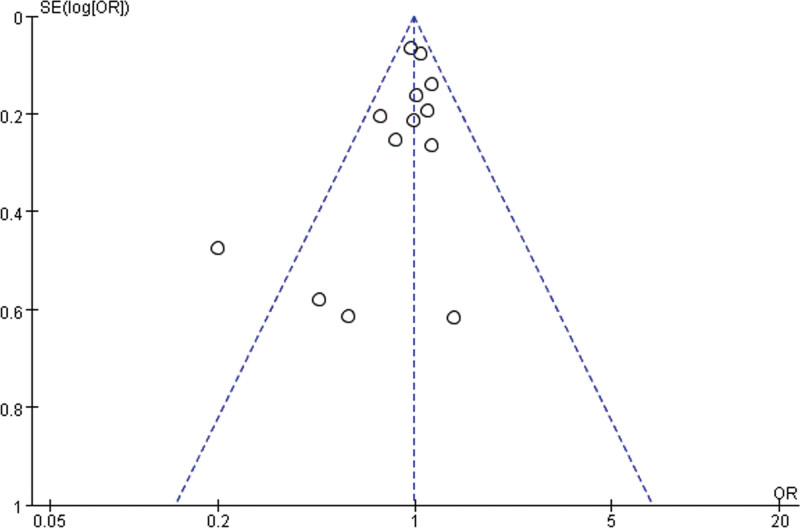
Adverse event funnel diagram.

In this meta-analysis, to investigate whether publication bias was present in the data related to ONJ and AEs, funnel plots were used for detailed analysis. From the results presented in the funnel plot, Figure 12 shows that the sample size is relatively small and the distribution on both sides is uneven, indicating a possible publication bias in the outcome index of ONJ. Figure 13 shows a more symmetrical distribution; however, one study was outside the graph, suggesting a possible publication bias in the outcome metrics of AEs.

In addition, this study conducted a sensitivity analysis of each outcome indicator to comprehensively assess the impact of each included study on the overall pooled data. The results showed that even when individual studies were excluded, the combined data did not change significantly, suggesting that the results of this study were more stable.

### 3.7. Evaluation of the quality of GRADE evidence

The quality of evidence related to outcomes was assessed in this study using the GRADE profiler software.^[30]^ Table 2 presents the GRADE evidence profile (the number of duplicate subjects in the same trial group has been removed). Due to heterogeneity and methodological issues, the hip BMD in this study was classified as very low-quality evidence. SAEs and lumbar spine BMD were moderate-quality evidence. The rest of the outcome metrics were low-quality evidence (in the penultimate column, one “⊕” and 3 “⊝” indicate “very low,” and 2 “⊕” and 2 “⊝” denote “low,” and 3 “⊕” and one “⊝” denote “very low.”) The poor quality of the available evidence suggests that the current evidence for the outcome metrics of denosumab in treating osteoporosis is of low quality and the conclusions are not highly credible. The low quality of the evidence may be due to the presence of many low-quality studies in the original literature. The methodological limitations of the original research significantly impacted the quality of evidence regarding the use of denosumab in treating osteoporosis, potentially leading to biased results. It is recommended that when researchers conduct relevant clinical RCTs in the future, they should follow the harmonized standards of the guidelines for reporting RCTs in order to control the quality of RCTs.

**Table 2:** GRADE evidence profile of denosumab group compared to conventional medications group for osteoporosis.

Denosumab vs conventional medications for osteoporosis						
Patient or population: patients with osteoporosis Settings: Intervention: denosumab Comparison: conventional medications						

Outcomes	Illustrative comparative risks* (95% CI)		Relative effect (95% CI)	No of participants (studies)	Quality of the evidence (GRADE)	Comments
	Assumed risk	Corresponding risk				
	Conventional medications	Denosumab				
Necrosis of the jaws Follow-up: mean 50.4 mo	Study population		OR 1.72 (0.7–4.2)	13,019 (5 studies)	⊕⊕⊝⊝ low^1^	
	38 per 1000	64 per 1000 (27–143)				
	Moderate					
	0 per 1000	0 per 1000 (0–0)				
Atypical fractures Follow-up: mean 52 mo	Study population		OR 1.77 (0.38–8.31)	12,373 (3 studies)	⊕⊕⊝⊝ low^1^	
	0 per 1000	1 per 1000 (0–3)				
	Moderate					
	1 per 1000	2 per 1000 (0–8)				
Adverse events Follow-up: mean 18.5 mo	Study population		OR 0.98 (0.91–1.07)	16,373 (13 studies)	⊕⊕⊝⊝ low^1^	
	797 per 1000	794 per 1000 (782–808)				
	Moderate					
	757 per 1000	753 per 1000 (739–769)				
Serious adverse events Follow-up: mean 19.6 mo	Study population		OR 0.94 (0.53–1.69)	16,126 (11 studies)	⊕⊕⊕⊝ moderate^1^	
	139 per 1000	132 per 1000 (79–215)				
	Moderate					
	64 per 1000	60 per 1000 (35–104)				
Lumbar Spine bone mineral density Follow-up: mean 24.8 mo		The mean spine bone mineral density in the intervention groups was 0.07 higher (0.03 to 0.11 higher)		16,437 (15 studies)	⊕⊕⊕⊝ moderate^1^	
Total hip bone mineral density Follow-up: mean 24.8 months		The mean total hip bone mineral density in the intervention groups was 0.03 higher (0 to 0.06 higher)		16,437 (15 studies)	⊕⊝⊝⊝ very low^1^	
		The mean femoral neck bone mineral density in the intervention groups was 0.02 higher (0.04 lower to 0.08 higher)		10,646 (10 studies)	⊕⊕⊝⊝ low^1^	

GRADE Working Group grades of evidence

High quality: Further research is very unlikely to change our confidence in the estimate of effect.

Moderate quality: Further research is likely to have an important impact on our confidence in the estimate of effect and may change the estimate.

Low quality: Further research is very likely to have an important impact on our confidence in the estimate of effect and is likely to change the estimate.

Very low quality: We are very uncertain about the estimate.

CI = confidence interval, OR = odds ratio.

* The basis for the assumed risk (e.g., the median control group risk across studies) is provided in footnotes. The corresponding risk (and its 95% confidence interval) is based on the assumed risk in the comparison group and the relative effect of the intervention (and its 95% CI).

### 3.8. TSA for SAEs

**Figure 14.**
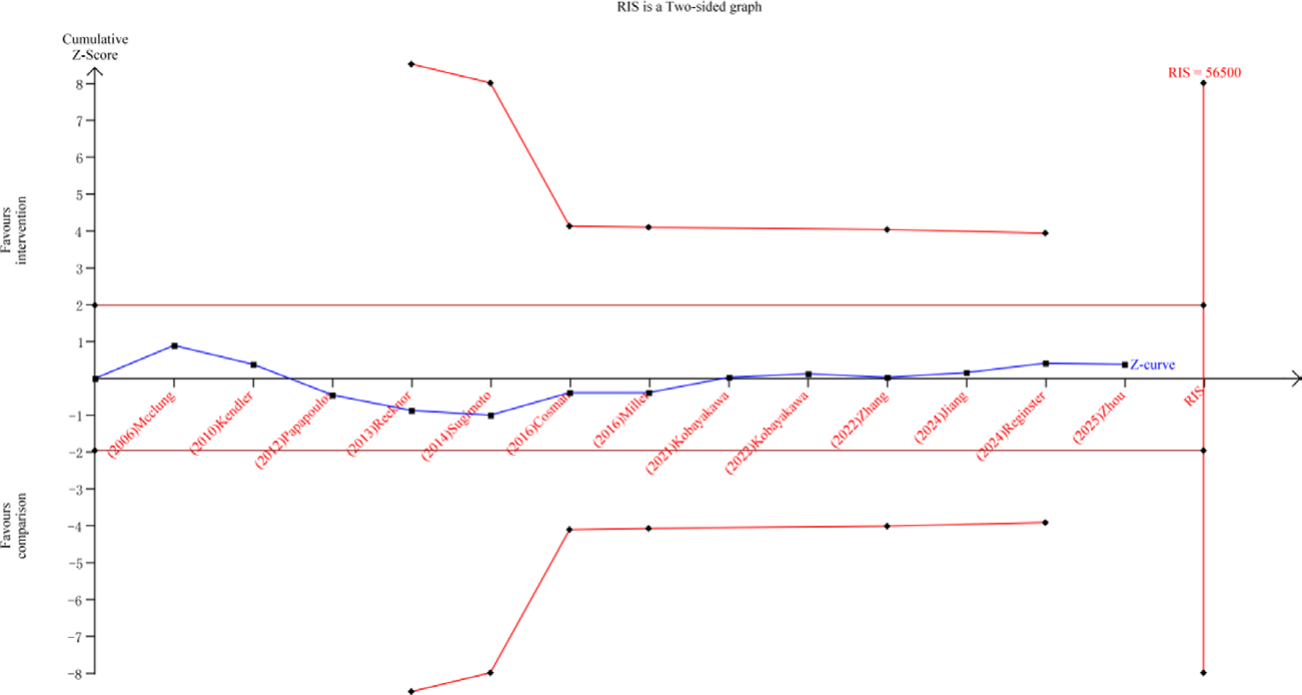
Trial sequential analysis for SAEs in the denosumab versus the conventional medications group for the treatment of primary osteoporosis. SAE = serious adverse event.

TSA was used to examine the reliability and conclusiveness of SAEs. In the current meta-analysis, the TSA was performed by maintaining 95% CIs with a control event rate of 20%, based on 13 studies included in this meta-analysis,^[16–26,28,29]^ a 1-sided a = 5% to minimize the possibility of type I error, and a statistical test power of 80%. TSA software version 0.9.5.10 Beta (Copenhagen Trial Unit; http://www.ctu.dk/tsa) was used in this study. If the cumulative Z-curve neither crossed the trial sequential monitoring boundary nor exceeded the required information size, a no difference result had been reached and no further studies were needed. In the TSA, our calculations determined that a sample size of 56,500 patients was necessary to detect a difference in SAEs. Additionally, the cumulative Z-curve did not cross the trial sequential monitoring boundary before surpassing the information size. This finding indicates that the cumulative evidence regarding the impact of denosumab on preventing osteoporosis-related SAEs is inconclusive (Fig. 14).

## 4. Discussion

Osteoporosis, a degenerative bone disease characterized by the progressive loss of bone microarchitecture and a decrease in bone density, leads to increased bone fragility and a heightened risk of fractures, particularly in older adults.^[31]^ This disease significantly affects quality of life, often leading to disability, morbidity, and even mortality in severe cases.

Current therapeutic strategies, including medications like denosumab,^[32]^ estrogen,^[33]^ calcitonin,^[34]^ and bisphosphonates,^[35]^ aim primarily to reduce bone resorption and restore bone mass.^[36]^ Bisphosphonates, for example, are widely used to inhibit osteoclast activity, while estrogen and calcitonin have roles in modulating bone turnover. Denosumab, a monoclonal antibody that targets RANKL, has emerged as an important alternative in managing osteoporosis due to its potent ability to inhibit osteoclast-mediated bone resorption, offering a potential edge over traditional therapies.

Given the increasing use of denosumab, particularly in postmenopausal women and other high-risk populations, it is essential to evaluate not only its clinical effectiveness but also its long-term safety profile. Numerous studies have confirmed denosumab’s efficacy in reducing the incidence of osteoporotic fractures and improving BMD in patients with osteoporosis.^[6,7,10]^ However, despite its clinical success, there are concerns about its potential adverse reactions, including hypocalcemia, infections, and, rarely, ONJ.^[12,15]^ These adverse effects necessitate careful patient monitoring and consideration when prescribing denosumab.

Moreover, the generalizability of findings related to denosumab’s efficacy and safety is vital. While clinical trials often provide robust evidence, real-world data, which include diverse patient populations and long-term follow-ups, are essential to better understand the full spectrum of its effects. For instance, studies have indicated that certain demographic groups, such as those in North America, Europe, Latin America, Australasia^[15]^ and Chinese,^[17]^ may experience different responses or adverse events. Therefore, further analysis of denosumab’s impact across various patient subsets and settings is critical to guide clinical decision-making.

By expanding the scope of research to include a broader range of clinical scenarios and by continuously monitoring safety profiles, we can better understand the balance between the therapeutic benefits and risks of denosumab in osteoporosis management. This approach not only ensures more personalized care but also contributes to the broader body of evidence needed to refine treatment guidelines and improve patient outcomes.

This meta-analysis compared the efficacy of denosumab and conventional medications for osteoporosis in 15 studies involving 16,437 patients with osteoporosis, divided into the denosumab group (n = 8506) and the conventional medication group (n = 7931). The denosumab group significantly increased the lumbar spine BMD of the subjects compared to the conventional drug group. Upon performing further sensitivity analyses, the evidence of benefit remained consistent. However, denosumab was not more beneficial in terms of increasing hip BMD and femoral neck BMD compared to the other medications. Additionally, it did not increase the risk of ONJ, atypical fractures, AEs, or SAEs, so our findings underscore the safety of denosumab regarding potential side effects in these domains. In the included studies, although the researches by Bone et al and Papapoulos et al belong to the same study system, the incidence of ONJ in Bone’s 10-year medication cycle study was significantly higher than that in Papapoulos’ 5-year stage study. This paper analyzes the possibility that denosumab may induce ONJ by continuously inhibiting osteoclast activity and bone remodeling, possibly synergizing with local trauma or infection factors, and that the risk accumulates with the prolongation of medication time. In addition, this meta-analysis revealed a high degree of heterogeneity in the results for lumbar spine BMD, hip BMD, and femoral neck BMD. Subgroup analyses of lumbar spine BMD revealed that medication duration may be a source of heterogeneity, and that further subgroup analyses of the outcome metrics will be necessary to identify possible sources of heterogeneity in the future.

Several studies looked into the effectiveness of denosumab with bisphosphonates in treating osteoporosis. Notably, Beaudoin et al and Lin et al evaluated the efficacy of denosumab versus bisphosphonates. Their findings revealed that the denosumab group witnessed a greater increase in bone density in the lumbar vertebrae, hip joints, femoral necks, and distal radius.^[37,38]^ The density comparison between the 2 groups, in terms of the degree of increase in bone density in other parts of the body, was not significant, which is inconsistent with the conclusions of this paper. However, this paper is not limited to comparing bisphosphonates as a class of medications, but also compares them simultaneously with the medications romosozumab and teriparatide, and the control group for the drug class is more comprehensive. Srivastava et al investigated the prevalence of drug-related ONJ in patients on sequential administration of anti-resorptive drug therapy.^[39]^ The prevalence of drug-related ONJ, for sequential bisphosphonate-denosumab therapy, the pooled weighted prevalence of ONJ was 13% (95% CI 3–22%) compared to only 5% (95% CI 0–9%) for bisphosphonates, and 4% (95% CI 3–5%) for denosumab only. In contrast, this paper directly compares the risk of ONJ after denosumab use with that of other medications, which can clarify the difference between denosumab and other medications in causing ONJ as an adverse effect. Additionally, it helps assess the safety profile of denosumab independently. In summary, the meta-analysis in this paper is the most recent and most comprehensive available.

As a result of the study in this article, it was found that the difference in clinical outcomes between the denosumab group and the control group was primarily due to the increase in BMD of the lumbar spine. Denosumab primarily works by specifically binding to the nuclear factor-κB receptor-activating protein ligand (RANKL), thereby preventing RANKL from binding to the RANK receptor on the cell surface of osteoclast precursor cells. This inhibition prevents the generation, function, and survival of osteoclasts, ultimately decreasing bone resorption and increasing bone density.^[40]^ While other medications such as bisphosphonates mainly act on osteoclasts, by inhibiting the activity of osteoclasts and inducing their apoptosis, thus reducing bone resorption;^[41,42]^ teriparatide mainly stimulates bone formation by binding to the PTH receptors on the surface of osteoblasts, activating a series of signaling pathways, and promoting osteoblasts’ proliferation, differentiation and activity.^[43]^ In general, an increase in bone density implies a decrease in the occurrence of fractures. Murad et al compared the effectiveness of different medications in preventing fragility fractures and found that teriparatide, bisphosphonates, and denosumab were the most effective in reducing the risk of fragility fractures; however, comparisons between each other were not statistically significant.^[44]^ This paper did not conduct a study on fracture risk and looks forward to the publication and meta-analysis of more clinical evidence on fractures in the future.

This review conducted an exhaustive search of central databases to identify all the RCTs examining denosumab for primary osteoporosis. Utilizing this strategy, common issues associated with non-randomized research, such as recall bias and selection bias, were addressed. Furthermore, 15 studies with a total of 16,437 patients were enrolled. Subgroup analyses were also performed to enhance the results and identify sources of heterogeneity. This comprehensive investigation not only reinforces the findings but also offers a more nuanced understanding of denosumab’s effectiveness in treating primary osteoporosis.

The current study pointed out numerous constraints. Most of the included studies had a treatment duration of 12 months. Given the relatively short treatment periods in the studies analyzed, it was not possible to determine the long-term safety of denosumab for osteoporosis. Some of the included articles had relatively small sample sizes, with their number of subjects ranging from a few dozen to several hundred. These tiny samples may be inadequate for correctly estimating treatment effects. A restricted sample size diminishes the statistical power to identify genuine differences or correlations between treatments and outcomes, thereby elevating the probability of false-positive and false-negative results. Searches for this study were limited to studies published in English or Chinese repositories. Therefore, potentially relevant RCTs published in languages other than English may have been missed, which could limit the generalizability of the findings of this study.

## 5. Conclusion

This meta-analysis evaluates the clinical effectiveness as well as safety of denosumab in comparison to standard treatments for primary osteoporosis. Denosumab is significantly superior to conventional medications in increasing lumbar spine BMD, and this finding has important implications for guiding clinical treatment strategies. It is noteworthy that, although denosumab showed no advantages in improving hip BMD and femoral neck BMD, the safety data indicated no statistically significant differences between the treatment and control groups in the incidence of ONJ, atypical fractures, AEs, and SAEs. However, limitations in this study still exist, which remind us that significant heterogeneity in the existing evidence may affect the reliability of the results. In the future, more high-quality, multicenter, large-sample, randomized double-blind RCTs are needed, with additional investigations on outcome indicators related to adverse reactions such as osteonecrosis of the jaw and atypical fractures, so as to establish a more reliable foundation for further research.

## Author contributions

**Conceptualization:** Liang Li.

**Data curation:** Jiahao Xu, Shuang Xia.

**Formal analysis:** Liang Li, Shuang Xia.

**Resources:** Liang Li.

**Software:** Liang Li.

**Writing – original draft:** Liang Li, Hongjian Ji.

**Writing – review & editing:** Hongjian Ji, Fengchao Shi.
